# Quality of MR thermometry during palliative MR-guided high-intensity focused ultrasound (MR-HIFU) treatment of bone metastases

**DOI:** 10.1186/s40349-015-0026-7

**Published:** 2015-03-24

**Authors:** Mie K Lam, Merel Huisman, Robbert J Nijenhuis, Maurice AAJ van den Bosch, Max A Viergever, Chrit TW Moonen, Lambertus W Bartels

**Affiliations:** Image Sciences Institute, University Medical Center Utrecht, Utrecht, The Netherlands; Department of Radiology, University Medical Center Utrecht, Utrecht, The Netherlands

**Keywords:** MR thermometry, Bone metastases, MR-HIFU, Treatment monitoring, PRFS

## Abstract

**Background:**

Magnetic resonance (MR)-guided high-intensity focused ultrasound has emerged as a clinical option for palliative treatment of painful bone metastases, with MR thermometry (MRT) used for treatment monitoring. In this study, the general image quality of the MRT was assessed in terms of signal-to-noise ratio (SNR) and apparent temperature variation. Also, MRT artifacts were scored for their occurrence and hampering of the treatment monitoring.

**Methods:**

Analyses were performed on 224 MRT datasets retrieved from 13 treatments. The SNR was measured per voxel over time in magnitude images, in the target lesion and surrounding muscle, and was averaged per treatment. The standard deviation over time of the measured temperature per voxel in MRT images, in the muscle outside the heated region, was defined as the apparent temperature variation and was averaged per treatment. The scored MRT artifacts originated from the following sources: respiratory and non-respiratory time-varying field inhomogeneities, arterial ghosting, and patient motion by muscle contraction and by gross body movement. Distinction was made between lesion type, location, and procedural sedation and analgesic (PSA).

**Results:**

The average SNR was highest in and around osteolytic lesions (21 in lesions, 27 in surrounding muscle, *n* = 4) and lowest in the upper body (9 in lesions, 16 in surrounding muscle, *n* = 4). The average apparent temperature variation was lowest in osteolytic lesions (1.2°C, *n* = 4) and the highest in the upper body (1.7°C, *n* = 4). Respiratory time-varying field inhomogeneity MRT artifacts occurred in 85% of the datasets and hampered treatment monitoring in 81%. Non-respiratory time-varying field inhomogeneities and arterial ghosting MRT artifacts were most frequent (94% and 95%) but occurred only locally. Patient motion artifacts were highly variable and occurred less in treatments of osteolytic lesions and using propofol and esketamine as PSA.

**Conclusions:**

In this study, the general image quality of MRT was observed to be higher in osteolytic lesions and lower in the upper body. Respiratory time-varying field inhomogeneity was the most prominent MRT artifact. Patient motion occurrence varied between treatments and seemed to be related to lesion type and type of PSA. Clinicians should be aware of these observed characteristics when interpreting MRT images.

**Electronic supplementary material:**

The online version of this article (doi:10.1186/s40349-015-0026-7) contains supplementary material, which is available to authorized users.

## Background

Magnetic resonance-guided high-intensity focused ultrasound (MR-HIFU) is a modality for non-invasive thermal therapy. Focused ultrasound is used to locally heat the tissue, while the treatment can be monitored real-time using MR thermometry (MRT). MR-HIFU has been used for tumor ablation in the bone [1-4], liver [2,3,5-13], pancreas [3,11,14], kidney [10,15], and breast [2,13,16-19]. Other clinical treatments that have been performed with MR-HIFU are the ablation of uterine fibroids [13,20-23] and of bone metastases for the purpose of pain palliation [13,24-28]. In this study, we focused on the palliative treatment of bone metastases with MR-HIFU. The pain mechanism is thought to be closely related to the periosteal innervation [25,26], and therefore, the aim is local periosteal denervation by heating the cortical bone. As cortical bone has a high acoustic absorption, the temperature in the bone will elevate more than that in the surrounding muscle tissue during exposure to HIFU [29]. Another advantage of treating painful bone metastases with MR-HIFU is the time to response of typically a few days [25,26,28], compared to weeks when using external beam radiotherapy, the current standard of practice [30,31]. Additionally, in contrast to external beam radiotherapy, MR-HIFU is not associated with radiation toxicity.

Unfortunately, the presence of the pain may complicate the treatment procedure. For example, the patient may not be able to lie still for a prolonged period of time or treatment-induced involuntary motion may occur if the treatment is not performed under general anesthesia. Patient motion may hamper the MR images that are used for treatment monitoring. Also, the image quality may be variable between specific cases for two reasons. First, bone metastases can occur at various locations. Second, there are three types of bone metastases: osteolytic, osteoblastic, and mixed. Osteolytic lesions are characterized by resorption of cortical bone, whereas osteoblastic lesions are characterized by formation of cortical bone. Mixed lesions exhibit both resorption and formation of cortical bone. Cortical bone has low water content [32] and a short *T*_2_ [33] and will thus give very low MR signal. Therefore, the image quality may possibly be different between lesion types.

The most commonly used method for temperature mapping in MR-HIFU, used for treatment monitoring, is based on the temperature-dependent proton resonance frequency shift (PRFS) [34,35]. Due to the lack of MR signal, PRFS-based MRT is unable to detect temperature changes in cortical bone. The bone marrow does give MR signal but has a high fat content. Since PRFS-based MRT only works in aqueous tissue, little to no temperature information can be retrieved from the bone marrow. The treatment monitoring during MR-HIFU of bone metastases is limited to the surrounding aqueous tissue. With PRFS-based MRT, temperature changes are calculated from phase differences obtained by phase image subtractions of gradient-echo scans [35]. The method is therefore sensitive to non-temperature-related spatiotemporal phase variations and subtraction errors, which will result in errors in the temperature images.

In this study, we evaluated the clinical performance of PRFS-based MRT used for monitoring of MR-HIFU ablation procedures of bone metastases that have been performed in our hospital. For this purpose, we assessed the general image quality by measuring the signal-to-noise ratio (SNR) and apparent temperature variations. Furthermore, potential artifacts in the temperature images were scored for their occurrence and hampering of treatment monitoring.

## Methods

### Ethics statement

Approval from the Institutional Review Board of the University Medical Center Utrecht (Utrecht, The Netherlands) was obtained for this study. All participants were counseled on the nature of the procedure, and all provided written informed consent for the treatment and use of their (anonymized) data.

### Patient characteristics

Eleven patients, referred to our hospital for clinical palliative treatment of metastatic bone pain after exhaustion of the standard of care, were treated with a clinical MR-HIFU platform (Sonalleve, Philips Healthcare, Helsinki, Finland), integrated into a clinical 1.5-T MRI scanner (Achieva, Philips, Best, The Netherlands). Two patients were retreated, resulting in 13 therapeutic sessions in total. Table 1 shows the patient characteristics. The treated lesions were located in the upper body (*n* = 4), the pelvis (*n* = 7), and in a lower extremity (*n* = 2). There were seven osteolytic lesions, five mixed lesions, and one osteoblastic lesion. Three types of intravenous procedural sedation and analgesia (PSA) were used: four patients received a combination of fentanyl (50–100 μg) and midazolam (2–5 mg) and will be referred to as PSA type A, four patients received propofol (induction 0.5–1 mg/kg, maintenance 5 mg/kg/h) combined with opioid analgesic at the discretion of the PSA specialist and will be referred to as PSA type B, and five patients received propofol (induction 0.5–1 mg/kg, maintenance 5 mg/kg/h) and esketamine as analgesic at the discretion of the PSA specialist and will be referred to as PSA type C. One patient treated in a lower extremity was retreated after 2 weeks at a different location and had metal internal fixation material in the target region. One patient treated in the pelvis was retreated after 4.5 months at the same location. A more detailed description of the treatments has been published elsewhere [36].

**Table 1:** **Description of the patient group**

**Treatment number**	**Sex**	**Age**	**Location**	**Lesion type**	**Receiver coil(s)**	**Number of datasets** ^**g**^
1^a,d^	M	58	Femur	Osteolytic	HIFU 3-elem	13
2^a,d^	M	58	Femur	Osteolytic	HIFU 3-elem	7
3^a^	F	55	Sacrum	Osteolytic	HIFU 5-elem	7
4^a^	F	56	Pubic bone	Mixed	HIFU 5-elem	11
5^b^	M	60	Pubic bone	Osteolytic	HIFU 5-elem	16
6^b,e^	F	64	Sacrum	Mixed	HIFU 5-elem	13
7^b^	F	53	Shoulder	Osteoblastic	MR Body coil	31
8^c^	M	86	Rib	Mixed	HIFU 5-elem	20
9^b,e,f^	F	64	Sacrum	Mixed	HIFU 5-elem	23
10^c,f^	M	55	Pubic bone	Osteolytic	HIFU 5-elem	27
11^c^	M	71	Pubic bone	Osteolytic	HIFU 5-elem	23
12^c^	M	65	Rib	Mixed	HIFU 5-elem	15
13^c^	M	64	Rib	Osteolytic	HIFU 5-elem	18
Total						224

^a^Performed under PSA type A (fentanyl and midazolam).

^b^Performed under PSA type B (propofol and opioid analgesic).

^c^Performed under PSA type C (propofol and esketamine).

^d^Same patient, retreated after 2 weeks, metal internal fixation material in the target region.

^e^Same patient, retreated after 4.5 months.

^f^Higher resolution MRT scans used.

^g^For each sonication, one dataset was acquired, containing a dynamic series of multi-slice magnitude images, phase images, and calculated temperature maps.

### MRI sequences

The built-in radio frequency (RF) receiver coil inside the HIFU window was used together with a HIFU pelvis RF receiver coil positioned on top of the patient. Patients were positioned with the target lesion above the transducer window in the MR-HIFU tabletop. The first two treatments were performed on an earlier version of the clinical MR-HIFU system (Sonalleve, Release 2), where the HIFU window coil consisted of one element and the HIFU pelvis coil of two elements. The remaining treatments were performed on the most recent version of the clinical MR-HIFU system (Sonalleve, Release 3), where the HIFU window coil had three elements and the HIFU pelvis coil had two. In the treatment of the metastasis in the shoulder, the patient did not fit into the bore with the pelvis receiver coil positioned on top due to the patient positioning and the built-in body coil of the MR scanner was used instead (Table 1).

**Figure 1:**
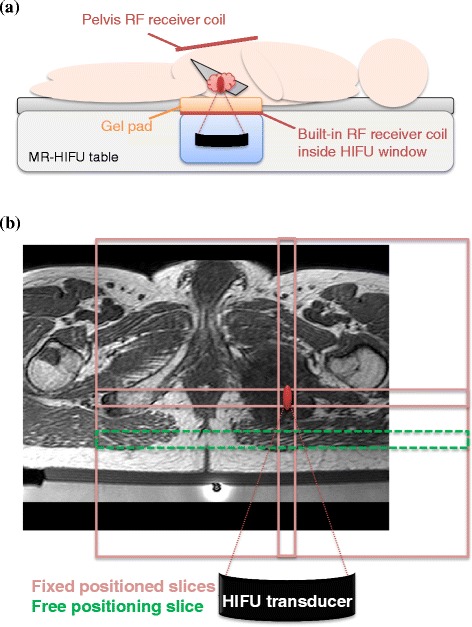
**Slice positioning of the MRT scans.** An example of an MR-HIFU setup for a treatment in the pelvis is shown in **(a)**. An example of the MRT scan slice positioning is shown in **(b)**, on a T1-weighted planning scan of an osteolytic lesion in the pubic bone (treatment 10). Three slices (light-red) were fixed with the centers to the location of the HIFU focus; one slice could be freely placed by the user and was placed in the near-field area of the HIFU beams (green, dashed).

Multi-planar reconstructed 3D T1-weighted spoiled gradient-echo scans were used for HIFU treatment planning with the following scan parameters: echo time = 4.6 ms, repetition time = 20 ms, flip angle = 30°, number of signal averages (NSA) = 2, number of slices = 100, field of view = 240 × 303 mm^2^, acquisition matrix = 184 × 201, acquired voxel size = 1.3 × 1.5 × 2.6 mm^3^. For HIFU treatment monitoring, a dynamic multi-slice, 2D spoiled gradient-echo echo-planar imaging (EPI) PRFS-based MRT sequence was used with water-selective binomial RF excitation pulses (1-2-1) with the following scan parameters: echo time = 19 ms, repetition time = 36 ms, flip angle = 20°, NSA = 2, EPI factor = 11, number of slices = 4, field of view = 400 × 310 mm^2^, acquisition matrix = 160 × 121, acquired voxel size = 2.5 × 2.6 × 7 mm^3^, and dynamic scan duration = 3.7 s. During two treatments, higher resolution PRFS-based MRT scans were used with the same scan parameters, except for NSA = 1, field of view = 400 × 307 mm^2^, acquisition matrix = 224 × 165, voxel size = 1.8 × 1.9 × 6.3 mm^3^, and dynamic scan duration = 2.7 s. The positions of three imaging slices (coronal, sagittal, transverse) were fixed, with the centers of the imaging slices positioned at the center of the HIFU focus location, as shown in Figure 1. A fourth imaging slice (coronal) was positioned in a muscular area closest to the transducer, also known as the near-field area of the HIFU beam (Figure 1). Two dynamics of the dynamic MRT scan were acquired before sonication, and images were acquired continuously up to 2 min of the total acquisition time.

### HIFU treatment

The MR-HIFU treatments were performed by volumetric sonications, where ellipsoidal volumes were treated by electronic steering of the HIFU focus in concentric circular trajectories of increasing diameter [37]. Treatment planning was done using the T1-weighted 3D scan. PRFS-based MRT images were used for temperature monitoring during the HIFU treatment. For each HIFU sonication, one dataset was obtained using an MRT pulse sequence, containing dynamic series of multi-slice magnitude images, phase images, and calculated temperature images. The temperatures were calculated by adding the patients’ baseline body temperature (auricularly measured) measured before treatment to the temperature differences derived from the phase images of the dynamic MRT data. No field drift correction was performed for the MRT data, since little drift was expected during the acquisitions (duration up to 2 min). Each HIFU treatment was preceded by one or more test sonications at low power (median 30 W, range 20–50 W) and with short duration (median 16 s, range 16–20 s). Therapeutic HIFU sonications were performed with variable power (median 95 W, range 10–160 W), variable duration (median 16 s, range 0.2–36 s), and variable cross-sectional diameter of the treatment volume (median 4 mm, range 2–12 mm) [37]. The acoustic power levels of the HIFU sonications were determined by the treating physician and could be selected up to the maximum power level allowed by the system, which ranged from 190 W for the smallest treatment volume to 80 W for the largest treatment volume. Data of both the test sonications and the therapeutic sonications were included. The total number of sonications within one treatment session ranged from 7 to 31, with a median of 16 (Table 1). A total number of 224 MRT datasets of 11 patients was used for the analysis, which included the datasets related to the test sonications.

### Data analysis: general image quality

The general image quality was assessed by measuring the signal-to-noise ratio in the magnitude images acquired with the PRFS sequence and the apparent temperature variation in the calculated temperature images.

An important indicator for general image quality is the SNR of the magnitude images of the datasets. To avoid the influence of tissue structure in SNR measurements, we measured the SNR in single voxels over time. For each dataset, two voxels were selected for each imaging slice: one in the target lesion region and one in a muscle region near the lesion region. The voxels were selected in the temperature image, away from the heated area and away from any obvious local artifacts. When muscle contraction and/or body movement was observed visually in the magnitude image, the whole dataset was excluded from the analysis. When the lesion region and/or the muscle region were not visible in an imaging slice, the slice was excluded from the analysis. The temporal mean and temporal standard deviation of the magnitude signal intensities in the selected voxels over all dynamics were determined. Subsequently, the SNR of each voxel was calculated by dividing the mean by the standard deviation. Per dataset, the SNR values of the voxels in the target lesion were averaged and the SNR values of the voxels in the surrounding muscle were averaged. Finally, the average SNR over all datasets in the target lesion and surrounding muscle was calculated per treatment.

As another measure of the general image quality, we measured the apparent temperature variation that was not influenced by heating or obvious artifacts. For each dataset, one voxel was selected for each slice in the temperature image in a muscle region, away from the heated area and away from any obvious local MRT artifact. The datasets that were excluded from the SNR analysis because muscle contraction and/or body movement were observed visually were also excluded from this analysis. When the muscle region was not visible in an imaging slice, the slice was excluded from the analysis. The apparent temperature variation was defined as the temporal standard deviation of the measured temperatures with PRFS-based MRT in the selected voxel over all dynamics. Per dataset, the apparent temperature variation values of the voxels were averaged. Finally, the average apparent temperature variation was calculated per treatment.

Qualitative comparisons were done between different lesion types (osteolytic, mixed, and osteoblastic) and locations (upper body, pelvis, and lower extremity). To make the comparisons as fair as possible, datasets were excluded of the treatments where metal fixation material was present and where higher resolution scans were used.

### Data analysis: artifacts

As PRFS-based temperature images are reconstructed from subtracted phase images [35], non-temperature-related phase changes will result in errors in the temperature images. From here on, these errors will be referred to as MRT artifacts. The dynamic multi-slice temperature images were scored by one observer (ML) for the occurrence of MRT artifacts and hampering of the treatment monitoring by MRT artifacts caused by the following sources: time-varying field inhomogeneities, arterial ghosting, and patient motion.

Field inhomogeneities are caused by the susceptibility distribution. Static field inhomogeneities will not lead to errors in temperature images, as they are canceled out by the subtraction of subsequent phase images. However, temporal changes of the susceptibility distribution will cause time-varying field inhomogeneities, leading to local non-temperature-related phase changes and resulting in MRT artifacts. Changing volumes of air is one of the most prominent sources of this type of artifact, as the susceptibility of air (*χ* = 0.36 ppm) differs considerably from that of human tissues (*χ* = −11.0 to −7.0 ppm) [38]. As the air volume in the lungs varies over the respiratory cycle, respiration can cause periodical phase variations in regions near the lungs [38]. Two categories of time-varying field inhomogeneity artifacts were distinguished: respiratory and non-respiratory. The respiratory MRT artifacts were classified as periodical temperature variations in the whole temperature map; the non-respiratory MRT artifacts as local highly variable temperatures near interfaces (e.g., bowel, rectum) and can be verified by looking at the phase images.

Arterial ghosting is caused by pulsatile blood flow, which leads to reconstruction of the MR signal of the blood at a different position than where it originated from [39]. In the phase image, this ghosting will appear as vessel-shaped areas with variable phase values, displacing over the image in the phase-encoding direction. The resulting MRT artifacts were classified as vessel-shaped objects with variable observed temperatures, displacing over the image in the phase-encoding direction.

Patient motion will lead to misregistration between subtracted phase images. Artifacts due to patient motion were scored as either due to muscle contraction or due to gross body movement. When both muscle contraction and gross body movement were observed, the artifact was scored as being caused by gross body movement. Classification was done by the observation of muscle contraction and gross body movement in the magnitude image; the resulting MRT artifacts were large observed temperature changes at the location and time of the motion. With gross body movement, the MRT artifact typically affected the whole temperature image. With muscle contraction, the MRT artifact occurred typically locally at the location of the muscle. However, due to the displacement of the tissue, non-respiratory time-varying field inhomogeneity artifacts may increase in size and severity. As the occurrence of patient motion may depend on different factors, distinction was made between lesion type, location, and PSA type.

An MRT artifact was scored as “occurred” when it was observed in at least one of the temperature imaging slices. The dataset was also scored as “hampered” if the visualization of the heat built-up due to the HIFU treatment and the following cooldown was distorted due to the MRT artifact. This could be observed as either temperature errors in and around the focus or the inability to detect (expected) HIFU heating: both may hamper the treatment monitoring. How often a type of MRT artifact occurred and/or hampered the treatment monitoring was determined per treatment and expressed as a percentage of the number of MRT datasets of the treatment, which will be referred to as the “occurrence rate” and the “hampering rate” from here on. Also, the total occurrence and total hampering of each artifact were determined and expressed as a percentage of all 224 MRT datasets, which will be referred to as the “total occurrence rate” and “total hampering rate”.

## Results

### General image quality

**Figure 2:**
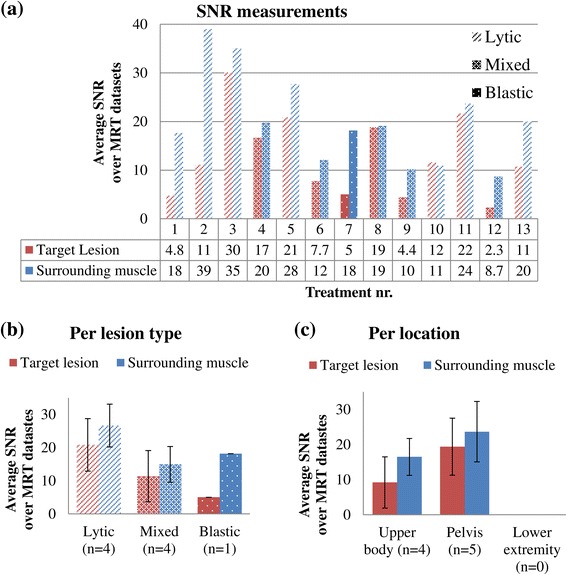
**The SNR measured in the magnitude images.** The average SNR is shown per treatment **(a)**, per lesion type **(b)**, and per location **(c)**. The rows with numbers below the graph in **(a)** show from top to bottom: treatment numbers, average SNR values in the target lesion, and average SNR values in the surrounding muscle. Treatment numbers 1, 2, 9, and 10 were excluded in **(b)** and **(c)**. The error bars in **(b)** and **(c)** represent the standard deviations.

Figure 2a shows the average SNR per treatment, distinction was made between the lesion types. The average SNR in the lesions ranged from 2.3 to 30 and in surrounding muscles from 8.7 to 39. Figure 2b shows the average SNR per lesion type and Figure 2c the average per location, where treatments 1, 2, 9, and 10 were excluded because of either the presence of a metal internal fixation material or the use of higher resolution scans. In the comparison between lesion types (Figure 2b), the highest average SNR was found in and around osteolytic lesions (lesions: 21 ± 8, surrounding muscles: 27 ± 6, *n* = 4). The average SNR in mixed lesions was 11 ± 8 and 15 ± 5 in surrounding muscles (*n* = 4); the average SNR in osteoblastic lesions was 5 and 18 in surrounding muscles (*n* = 1). In the comparison between locations (Figure 2c), the average SNR was higher in the pelvis (lesions: 19 ± 8, surrounding muscles: 24 ± 9, *n* = 5), as compared to the upper body (lesions: 9 ± 7, surrounding muscles: 16 ± 5, *n* = 4). Because of the exclusion of the treatments with metal fixation material present, there were no datasets left in the lower extremity region.

**Figure 3:**
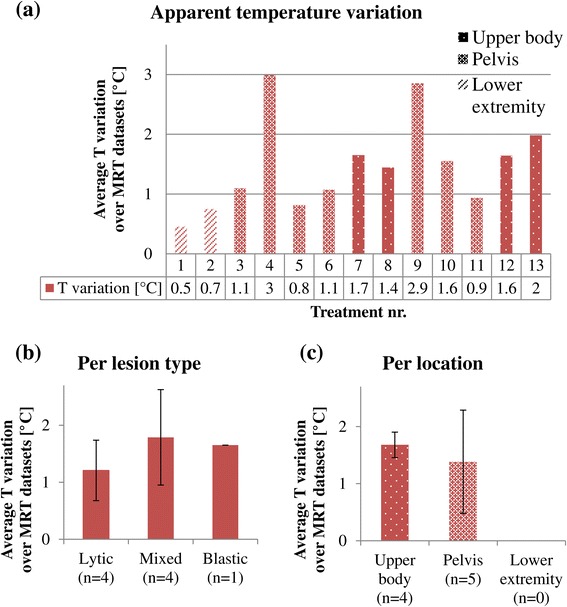
**The apparent temperature variation measured in the temperature images.** The average temperature variation is shown per treatment **(a)**, per lesion type **(b)**, and per location **(c)**. The rows with numbers below the graph in **(a)** show the treatment numbers (top) and average apparent temperature variation values (bottom). Treatment numbers 1, 2, 9, and 10 were excluded in **(b)** and **(c)**. The error bars in **(b)** and **(c)** represent the standard deviations.

Figure 3a shows the average apparent temperature variation per treatment, distinction was made between the locations. The apparent temperature variation ranged from 0.5°C to 3°C. Treatments 4 and 9 are the only two with a variation larger than 2°C and were both mixed lesions in the pelvis. Similar to the SNR analysis, treatments 1, 2, 9, and 10 were excluded in the comparisons between lesion types and locations. In the comparison between lesion types (Figure 3b), the apparent temperature variation in the datasets of the osteolytic lesion (1.2 ± 0.5°C, *n* = 4) was found to be lower than in the datasets of the mixed (1.8°C ± 0.8°C, *n* = 4) and osteoblastic lesions (1.7°C, *n* = 1). In the comparison between locations (Figure 3c), the apparent temperature variation was found to be higher in the upper body (1.7°C ± 0.2°C, *n* = 4) compared to the pelvis (1.4°C ± 0.9°C, *n* = 5). Because of the exclusion of the treatments with metal fixation material present, there were no datasets left in the lower extremity region.

### Artifacts

**Figure 4:**
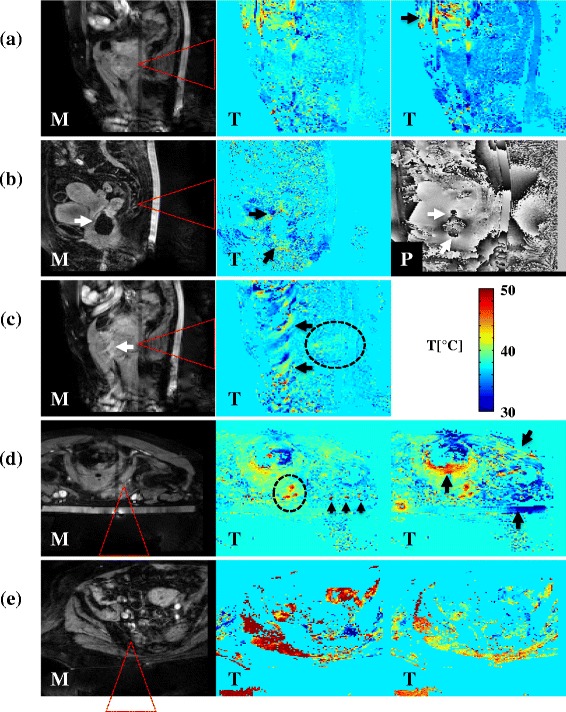
**Typical examples of MRT artifacts.** The arrows point out MRT artifacts in magnitude (M) images, temperature (T) images, and phase (P) images. The red dashed triangles indicate the expected HIFU cone. **(a)** A respiratory time-varying field inhomogeneity MRT artifact (treatment 5, sagittal slice, supine position, osteolytic lesion in the pubic bone). The artifact causes periodical “blinking” of the temperature map. The arrow points out an additional non-respiratory time-varying field inhomogeneity MRT artifact. **(b)** A non-respiratory time-varying field inhomogeneity MRT artifact (treatment 9, sagittal slice, supine position, mixed lesion in the sacrum). The artifact is caused by an air cavity, which can also be seen in the magnitude image. The local changes in the phase image around the location of the air cavity verify that the air cavity is the source. **(c)** An arterial ghosting MRT artifact (treatment 5, sagittal slice, supine position, osteolytic lesion in the pubic bone) caused by the femoral artery, which can also be seen in the magnitude image. **(d)** Muscle contraction MRT artifacts (treatment 5, transverse slice, prone position, mixed lesion in the pubic bone). The artifacts occur not only at the location of the contracting muscles (two arrows at the most right) but also around the rectum. The three arrowheads point out additional arterial ghosting MRT artifacts. **(e)** A gross body movement MRT artifact (treatment 6, transverse slice, supine position, mixed lesion in the sacrum, affects the whole temperature image drastically. In the dashed ellipses in **(c)** and **(d)**, heating due to the HIFU treatment can be observed. The image shown in **(b)** was acquired before HIFU sonication started; thus, no HIFU heating was expected to be observed. In **(a)** and **(e)**, the visualization of potential HIFU heating was hampered due to the presence of the artifact.

Typical examples of the MRT artifacts that were scored are shown in Figure 4, together with the corresponding magnitude images for the visualization of the anatomy: Figure 4a shows a respiratory time-varying field inhomogeneity MRT artifact, a movie of the dynamic temperature map can be found in Additional file 1; Figure 4b shows a non-respiratory time-varying field inhomogeneity MRT artifact, of which the origin of the artifact could be verified in the phase image; Figure 4c shows a typical arterial ghosting MRT artifact; Figure 4d shows patient motion MRT artifacts due to muscle contraction; and Figure 4e shows patient motion MRT artifacts due to gross body movement.

**Figure 5:**
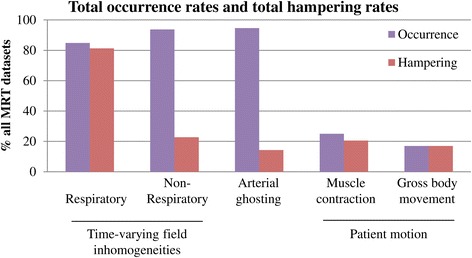
**Total occurrence rates and total hampering rates of MRT artifacts.** The occurrence rates and hampering rates are shown per source, in % of all MRT datasets.

Figure 5 shows the total occurrence of and hampering of the treatment monitoring by MRT artifacts in percentage of all datasets of all treatments, per source. It can be seen that when artifacts occurred due to respiratory time-varying field inhomogeneities or patient motion, the artifacts hampered the visualization of the heat built-up in most cases. MRT artifacts due to non-respiratory time-varying field inhomogeneities and arterial ghosting were observed in almost all datasets, but only few have hampered the treatment monitoring.

The occurrence rates and hampering rates of the MRT artifacts per treatment are shown in Table 2 as percentage of the total number of datasets. The total occurrence rate of respiratory time-varying field inhomogeneity MRT artifact was 85%, and the total hampering rate was 81%. This artifact did not occur in the two treatments in the lower extremity, while in 8 treatments, the occurrence rate was 100%. The total occurrence rate of non-respiratory time-varying field inhomogeneity MRT artifact was 94%, and the total hampering rate was 23%. This artifact did not occur in one treatment but occurred 97% in one treatment and 100% in the remaining treatments. The total occurrence rate of arterial ghosting MRT artifacts was 95%, and the total hampering rate was 14%. This artifact did not occur in one treatment but occurred more than 80% in the remaining treatments, of which in 10 treatments the occurrence rate was 100%. The total occurrence rate of muscle contraction MRT artifact was 25%, and the total hampering rate was 21%. This artifact occurred in all treatments but less than 20% in 7 treatments. The total occurrence rate of gross body movement MRT artifact was 17%, and the total hampering occurrence rate was 17%.

**Table 2:** **Occurrence rates and** ***hampering rates*** **of the MRT artifacts in % of the total number of datasets per treatment**

**Treatment number**	**Location**	**Lesion type**	**Time-varying field inhomogeneities**				**Arterial ghosting**		**Patient motion**			
			**Respiratory**		**Non-Respiratory**				**Muscle contraction**		**Body movement**	
1^a,d^	Femur	Osteolytic	0	*0*	0	*0*	100	*0*	15	*0*	0	*0*
2^a,d^	Femur	Osteolytic	0	*0*	100	*0*	100	*0*	14	*0*	0	*0*
3^a^	Sacrum	Osteolytic	100	*100*	100	*0*	0	*0*	43	*29*	0	*0*
4^a^	Pubic bone	Mixed	82	*64*	100	*82*	100	*82*	91	*73*	0	*0*
5^b^	Pubic bone	Osteolytic	69	*38*	100	*0*	100	*0*	6.3	*6.3*	13	*13*
6^b,e^	Sacrum	Mixed	100	*100*	100	*0*	100	*0*	38	*31*	54	*54*
7^b^	Shoulder	Osteoblastic	100	*100*	97	*9.7*	100	*3.2*	39	*35*	35	*35*
8^c^	Rib	Mixed	100	*100*	100	*0*	100	*20*	10	*10*	0	*0*
9^b,e,f^	Sacrum	Mixed	100	*100*	100	*0*	83	*0*	17	*17*	39	*39*
10^c,f^	Pubic bone	Osteolytic	100	*100*	100	*33*	100	*0*	3.7	*3.7*	3.7	*3.7*
11^c^	Pubic bone	Osteolytic	70	*65*	100	*22*	96	*35*	22	*17*	13	*13*
12^c^	Rib	Mixed	100	*100*	100	*100*	100	*67*	53	*47*	33	*33*
13^c^	Rib	Osteolytic	100	*100*	100	*56*	100	*0*	11	*11*	0	*0*
Total			85	*81*	94	*23*	95	*14*	25	*21*	17	*17*

The occurrence rates are shown in normal font, the hampering rates in italic font.

^a^Performed under PSA type A (fentanyl and midazolam).

^b^Performed under PSA type B (propofol and opioid analgesic).

^c^Performed under PSA type C (propofol and esketamine).

^d^Same patient, retreated after 2 weeks, metal internal fixation material in the target region.

^e^Same patient, retreated after 4.5 months.

^f^Higher resolution MRT scans used.

**Figure 6:**
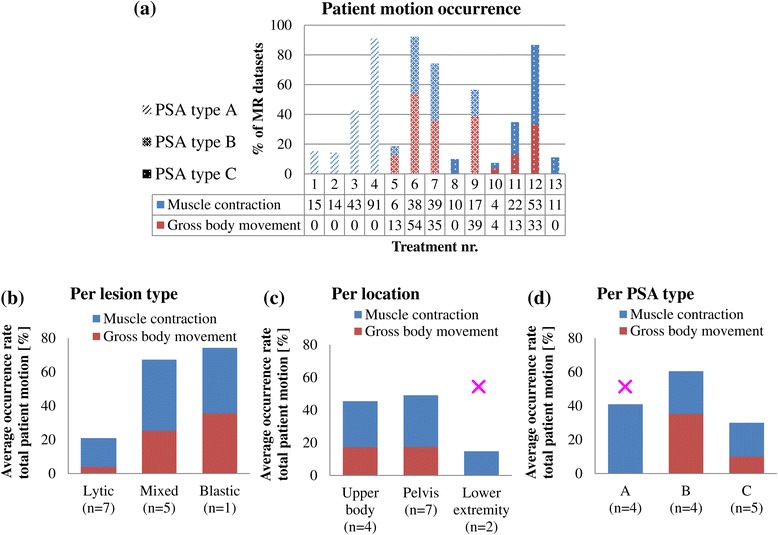
**Overview of the patient motion MRT artifacts occurrence rates.** The patient motion occurrence rates are shown in % of all MRT datasets. **(a)** The patient motion occurrence rates per treatment. The average occurrence rates are shown per lesion type **(b)**, per location **(c),** and per PSA type **(d)**. PSA type A: fentanyl and midazolam, PSA type B: propofol and opioid analgesic, PSA type C: propofol and esketamine. The rows with numbers below the graph in **(a)** show from top to bottom: treatment numbers, patient motion occurrence rates in % due to muscle contraction, and patient motion occurrence rates in % due to gross body movement. Please note that the lower extremity treatments were only performed using sedation A (indicated with the pink ×).

The variation of patient motion occurrence rate between treatments is visualized in Figure 6a, where distinction was made between PSA types. In some treatments, almost no patient motion occurred, while in some treatments, patient motion was dominantly present. Figure 6b shows the comparison between lesion types, and the lowest rates were found in osteolytic lesion types. The total patient motion occurrence rate was 20% (16% muscle contraction, 4% gross body movement, *n* = 7) in the osteolytic lesion datasets as compared to the 67% (42% muscle contraction, 25% gross body movement, *n* = 5) in the mixed lesion datasets and 74% (39% muscle contraction, 35% gross body movement, n = 1) in the osteoblastic lesions datasets. Figure 6c shows the comparison between locations: the total patient motion occurrence rate was similar for the upper body (45% in total: 28% muscle contraction, 17% gross body movement, *n* = 4) and the pelvis (49% in total: 32% muscle contraction, 17% gross body movement, *n* = 7) and lowest in the lower extremity (15% in total: 15% muscle contraction, 0% gross body movement, *n* = 2). The total patient motion occurrence rates are compared between PSA types in Figure 6d. For PSA type A, the total patient motion occurrence rate was 41% (41% muscle contraction, 0% gross body movement, *n* = 4); please note that this group includes the two lower extremity treatments. The total patient motion occurrence rate was higher for PSA type B (60% in total: 25% muscle contraction, 35% gross body movement, *n* = 4) as compared to PSA type C (30% in total: 20% muscle contraction, 10% gross body movement, *n* = 5).

## Discussion

The quality of the MR thermometry used for the monitoring of 13 palliative treatment patients with painful bone metastases with MR-HIFU was assessed in terms of general image quality and artifacts.

### General image quality

The comparison between lesion types shows descending average SNR in the target lesion with increasing amount of cortical bone (osteolytic: 21, mixed: 11, osteoblastic: 5). The average SNR in the surrounding muscle was highest around osteolytic lesions (27) and was similar for the mixed and osteoblastic type (15 compared to 18). The apparent temperature variation was high in the mixed lesions (1.8°C) and osteoblastic lesions (1.7°C) and low in the osteolytic lesions (1.2°C). The descending SNR in the lesions could be explained by the difference in the amount of cortical bone present. Cortical bone has a very short *T*_2_* [33] and will thus give very little signal in PRFS-based MRT sequences, which need a relatively long echo time. It was therefore expected that osteoblastic lesions will have a lower SNR compared to osteolytic lesions. However, no differences in SNR in the surrounding muscle would be expected between lesion types. There are several possible explanations for the observed differences in SNR in the surrounding muscle. First, in two treatments of mixed lesions, the apparent temperature variations were larger than 2°C, implying a relatively large influence of respiration effects in the measured SNR values. Second, only one osteoblastic lesion was treated in this study. For this treatment, the MR body coil was used for the image acquisition, while the dedicated HIFU coil combination was used in all other treatments.

The comparison between locations showed lower SNR in the upper body (9 in lesion, 16 in surrounding muscle) than in the pelvis (19 in lesion, 24 in surrounding muscle). The smallest apparent temperature variation was found in the lower extremity (0.6°C), where no respiratory time-varying field inhomogeneity artifacts were observed. The apparent temperature variation was higher in the upper body (1.7°C) as compared to in the pelvis (1.4°C). The standard deviation of the apparent temperature variation in the pelvis was relatively large (0.9°C), which can be explained by the fact that this group contained the two outliers with an apparent temperature variation larger than 2°C (treatment numbers 4 and 9). Please note that these outliers were both mixed lesion in the pelvis. Both the SNR and apparent temperature variation measurements indicate an increasing image quality with increasing distance to the upper body. This has also been observed previously by Peters et al. [40], in their measurements of respiratory field changes in the breast of volunteers using a dedicated scan sequence to investigate the effects of respiration. They reported maximum field fluctuations values over time during regular respiration of 0.13 ppm, corresponding to 13°C, which is much larger than the observed temperature variations in this study. The discrepancy can be explained by two differences between the study by Peters et al. and our study. First, we measured respiration-induced fluctuations over time, while Peters et al. measured fluctuation values that were spatially averaged over a region of interest covering both breasts; average fluctuations over time were not reported. Second, the dynamic scan duration in the study of Peters et al. was 0.64 s, while it was 3.7 s in our study. Also, the MRT sequence in our study was a segmented EPI, with 11 segments per *k*-space. In the presence of respiration of which the period is in the same order as the dynamic scan duration, the different *k*-space segments may have been affected differently by the field offsets induced by the respiration. Therefore, respiration-induced fluctuations may appear differently than in a non-segmented EPI scan, such as spoiled gradient-echo scan [40].

Voxels were selected carefully for the assessment of the general image quality, such that influences of HIFU heating, patient motion, and obvious local artifacts in the MR images were avoided. No corrections were made for the respiration. Since variations due to respiration were observed in the majority of the datasets (85%), exclusion of affected datasets would result in too few datasets for analysis. Frequency analysis could be an alternative way to filter out respiration effects. However, the datasets contained few samples (16 time points on average) and had a low sampling frequency (once per 3.7 s, corresponding to about 0.3 Hz). The respiration rate was not measured during the treatment. The range of respiration rates in adults is 12 to 18 per minute [41], corresponding to 0.2–0.3 Hz, which cannot be resolved with the current sampling. Also, we observed that the breathing pattern was typically irregular in both amplitude and frequency during the treatment, making accurate frequency analyses even more difficult. For these reasons, filtering of the respiration effects by frequency analysis was deemed not feasible in this study. Therefore, the SNR and apparent temperature variation measurements in this study represent the image quality (degradation) due to noise and variations induced by the respiration. Although the respiration effects could not be filtered or corrected, these measurements can still be used as a rough estimate of the image quality for the purpose of treatment monitoring. Because the SNR was measured in the magnitude images, it predominantly represents the image quality in terms of noise. Similarly, because the apparent temperature variations were measured in the PRFS-based temperature images, they predominantly represent the image degradation due to respiration.

**Figure 7:**
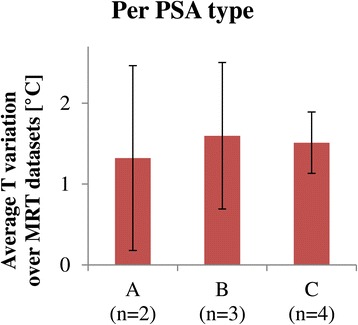
**Apparent temperature variation, averaged per PSA type.** PSA type **A**: fentanyl and midazolam, PSA type **B**: propofol and opioid analgesic, PSA type **C**: propofol and esketamine. Treatment numbers 1 and 2 were excluded due to the presence of metal internal fixation material, and treatment numbers 9 and 10 were excluded due to the use of higher resolution scans.

Recently, Deckers et al. [42] reported the effects of sedation on the respiration and the thermometry quality in four patients for MR-HIFU ablation of breast cancer. They observed that the use of propofol and esketamine (PSA type C in this study) resulted in more shallow and regular breathing patterns of the patients during treatment as compared to the use of propofol and opioid analgesic (PSA type B in this study). In this study, no reduction of the apparent temperature variation was measured with the use of propofol and esketamine (Figure 7). As the apparent temperature variation predominantly represented the image degradation due to respiration, this implies no regularization of the breathing pattern with the use of propofol and esketamine. The study reported by Deckers et al. [42] was performed on patients with breast cancer; the patients of this study were patients with bone metastases in an advanced stage of their disease. The purpose of the MR-HIFU treatment of these patients was to palliate the pain that could not be reduced sufficiently by the standard of care, often including opioid analgesics. As a consequence of their history of opioid usage, the patients included in our study may have reacted differently to the same type of PSA than patients with breast cancer who were typically opioid-naive.

The observations made in this study suggest that in MR-HIFU treatments of patients with bone metastases, the general MRT image quality is related to the lesion type and location. However, no statistical analyses were performed due to the small sample size and heterogeneous patient population, which limit the validity of the outcome. To establish and further quantify this association, a larger dataset is needed.

### Artifacts

The most dominant MRT artifact was the respiratory time-varying field inhomogeneity MRT artifact (85% occurrence rate, 81% hampering rate). Although the non-respiratory time-varying field inhomogeneity artifacts and arterial ghosting occurred more often (94% and 95%), these artifacts hampered the treatment monitoring only in a small number of cases (23% and 14%). This difference can be explained by the fact that non-respiratory time-varying field inhomogeneities and arterial ghosting induce local MRT artifacts, whereas respiratory time-varying field inhomogeneities induce large spatial field gradients, and thereby, temperature offsets potentially in the whole field of view. The total occurrence of and hampering of the treatment monitoring by patient motion MRT artifacts, both muscle contraction and gross body movement, was low compared to the other artifacts.

The patient motion occurrence rates of the individual treatments show that there is a large variation between treatments. Interestingly, patient motion occurred less in the datasets of the osteolytic lesions (14%) as compared to the mixed (61%) and osteoblastic lesions (70%). This observation suggests a relation between the presence of cortical bone in the lesion and patient motion. If the presence of bone implies the presence of periosteal nerves, this relation could possibly be explained by (involuntary) motion induced by periosteal nerve stimulation. In the comparison between locations, the average occurrence rate was lowest in the lower extremity, which is likely easier to control in terms of motion as compared to the pelvis and the upper body. In the comparison between PSA types, type A contained the two treatments in the lower extremity and may therefore be biased. The average occurrence rate was lower for PSA type C (30%) as compared to PSA type B (60%). This observation is in the same line as the observed regularization of the breathing pattern by Deckers et al. [42], with the use of propofol and esketamine (PSA type C) as compared to propofol and opioid analgesic (PSA type B). However, as the sample size is small and the patient population is heterogeneous, more data is required to further investigate the potential relations found in this study.

### Future prospects

Although the analyzed data was of a small patient group, a large number of datasets was analyzed and we have made observations possibly allowing improvements for future MR-HIFU treatments of bone metastases.

Measurement of the SNR and the apparent temperature variation could serve as a rough estimator of the general image quality. Higher SNR was observed in osteolytic lesions, and the apparent temperature variations were observed to decrease as the distance to the lungs increased. The observations made in this study should be kept in mind, as they could indicate a potential dependency of the expected image quality on the lesion types and/or location.

The MRT artifact analysis revealed the highest occurrence rate for non-respiratory time-varying field inhomogeneity artifacts and arterial ghosting. Although the direct hampering of the heat built-up visualization was minimal, the presence of the artifacts may cause problems when the temperature maps are automatically post-processed before they are shown to the clinician. For example, a post-processing algorithm could be used to classify observed temperature changes as apparent or true temperature changes and mask out apparent temperature changes. However, if the classification is based on observed temperature changes in a region where local MRT artifacts occur, these local MRT artifacts will indirectly hamper the treatment monitoring. Placement of a saturation slab can be considered to remove local artifacts. In case arterial ghosts do hamper the visualization of the heat built-up, changing the phase encoding direction could be considered so that the ghosting direction will be changed as well. In all cases, the adjustments should be made case-specific and care should be taken when interpreting the MR temperature images. Recently, an advanced spatiotemporal filtering post-processing method was proposed to remove MRT artifacts due to air bubbles in the rectum and was tested retrospectively [43]. This technique has potential to improve the MRT quality in real-time for treatment monitoring but requires further investigation before it can be applied in clinical practice.

The occurrence rate of patient motion was variable, and two important observations were made: the occurrence rate was lower in the treatments of osteolytic lesions and the occurrence rate was lower with the use of PSA type C (propofol and esketamine). Although the number of treatments included in this study was small, these observations should be kept in mind for future MR-HIFU treatments of bone metastases.

Respiratory time-varying field inhomogeneity artifacts were found to occur and hamper the treatment monitoring in the majority of the cases. This “blinking artifact” makes it challenging to determine the actual temperature increase due to HIFU heating. Nevertheless, the MRT used in this study allows localization of the heat built-up, which is the most important aspect of the treatment monitoring of the pain palliative treatment of bone metastases. However, for the other (potential) MR-HIFU applications in bone (metastases), i.e., tumor control by ablation [1-4] and local drug delivery [44,45], accurate temperature measurements are necessary. For total tumor ablation, a lethal thermal dose should be reached in the whole tumor without damaging the surrounding healthy tissue and thus reliable temperature measurements are required. In the pre-clinical local drug delivery study by Staruch et al. [44], it was shown that doxorubicin encapsulated in temperature-sensitive liposomes could be released *in vivo* in femurs of rabbits by MR-HIFU-induced mild hyperthermia. The release temperature window is typically between 41°C and 43°C and this mild hyperthermia was achieved and controlled by means of a feedback algorithm, using the real-time MR temperature information [46]. However, temperature errors due to the presence of MRT artifacts may affect the feedback algorithm and impede the temperature control. If tumor ablation and local drug delivery are to be applied in the patient population reported in this study, advanced methods to compensate for the respiration [47,48] and more sophisticated MR thermometry methods will be necessary, as these applications require more than solely localization of the heat deposition [49-51].

## Conclusion

The image quality of PRFS-based temperature images used for monitoring MR-HIFU palliative treatments of bone metastases was assessed. The general image quality was variable and was observed to be better in osteolytic lesions as compared to other lesion types and worse in the upper body as compared to the pelvis, in terms of SNR and apparent temperature variation. However, more treatment data is required to verify these potential relations. The MRT images were scored for the occurrence of MRT artifacts and hampering of treatment monitoring by MRT artifacts. Respiratory time-varying field inhomogeneity MRT artifacts were the most dominant and were also observed in the pelvic area, which is rather distal from the upper body. The MRT artifacts with the highest occurrence rate were those induced by non-respiratory time-varying field inhomogeneities and arterial ghosting. But because these were local artifacts, the hampering rate was low. The occurrence rate of patient motion was variable between treatments and seemed to be related to the presence of cortical bone in the lesion. Lower occurrence rates were observed with the use of propofol and esketamine as compared to the other PSA types. Clinicians should be aware of these artifacts and interpret the MRT images carefully when used for monitoring MR-HIFU treatments in bone.

## Additional file

Additional file 1:
**Movie: typical example of respiratory time-varying field inhomogeneity artifact.** To visualize the respiratory time-varying field inhomogeneity artifact more clearly, the dynamic temperature maps of the dataset shown in Figure 4a are shown here as a movie. The typical periodical “blinking” of the temperature map can be observed, most prominently between the 2nd and 3rd frame. Please note that in the top left a non-respiratory time-varying field inhomogeneity artifact is visible, as was pointed out in Figure 4. Also, arterial ghosting artifacts caused by the femoral artery can be seen as a blinking vertical stripe in the middle of the map.

## References

[1] Li C, Zhang W, Fan W, Huang J, Zhang F, Wu P (2010). Noninvasive treatment of malignant bone tumors using high-intensity focused ultrasound. Cancer.

[2] Wu F, Chen WZ, Bai J, Zou JZ, Wang ZL, Zhu H (2001). Pathological changes in human malignant carcinoma treated with high-intensity focused ultrasound. Ultrasound Med Biol.

[3] Orgera G, Monfardini L, Della Vigna P, Zhang L, Bonomo G, Arnone P (2011). High-intensity focused ultrasound (HIFU) in patients with solid malignancies: evaluation of feasibility, local tumour response and clinical results. Radiol Med.

[4] Chen W, Zhu H, Zhang L, Li K, Su H, Jin C (2010). Primary bone malignancy: effective treatment with high-intensity focused ultrasound ablation. Radiology.

[5] Leslie T, Ritchie R, Illing R, Ter Haar G, Phillips R, Middleton M (2012). High-intensity focused ultrasound treatment of liver tumours: post-treatment MRI correlates well with intra-operative estimates of treatment volume. Br J Radiol.

[6] Kennedy JE, Wu F, ter Haar GR, Gleeson FV, Phillips RR, Middleton MR (2004). High-intensity focused ultrasound for the treatment of liver tumours. Ultrasonics.

[7] Wu F, Wang ZB, Chen WZ, Zou JZ, Bai J, Zhu H (2005). Advanced hepatocellular carcinoma: treatment with high-intensity focused ultrasound ablation combined with transcatheter arterial embolization. Radiology.

[8] Zhu H, Zhou K, Zhang L, Jin C, Peng S, Yang W (2009). High intensity focused ultrasound (HIFU) therapy for local treatment of hepatocellular carcinoma: role of partial rib resection. Eur J Radiol.

[9] Xu G, Luo G, He L, Li J, Shan H, Zhang R (2011). Follow-up of high-intensity focused ultrasound treatment for patients with hepatocellular carcinoma. Ultrasound Med Biol.

[10] Illing RO, Kennedy JE, Wu F, ter Haar GR, Protheroe AS, Friend PJ (2005). The safety and feasibility of extracorporeal high-intensity focused ultrasound (HIFU) for the treatment of liver and kidney tumours in a Western population. Br J Cancer.

[11] Jung SE, Cho SH, Jang JH, Han JY (2011). High-intensity focused ultrasound ablation in hepatic and pancreatic cancer: complications. Abdom Imaging.

[12] Zhang Y, Zhao J, Guo D, Zhong W, Ran L (2011). Evaluation of short-term response of high intensity focused ultrasound ablation for primary hepatic carcinoma: utility of contrast-enhanced MRI and diffusion-weighted imaging. Eur J Radiol.

[13] Napoli A, Anzidei M, Ciolina F, Marotta E, Cavallo Marincola B, Brachetti G (2013). MR-guided high-intensity focused ultrasound: current status of an emerging technology. Cardiovasc Intervent Radiol.

[14] Wu F, Wang ZB, Zhu H, Chen WZ, Zou JZ, Bai J (2005). Feasibility of US-guided high-intensity focused ultrasound treatment in patients with advanced pancreatic cancer: initial experience. Radiology.

[15] Ritchie RW, Leslie T, Phillips R, Wu F, Illing R, ter Haar G (2010). Extracorporeal high intensity focused ultrasound for renal tumours: a 3-year follow-up. BJU Int.

[16] Hynynen K, Pomeroy O, Smith DN, Huber PE, McDannold NJ, Kettenbach J (2001). MR imaging-guided focused ultrasound surgery of fibroadenomas in the breast: a feasibility study. Radiology.

[17] Gianfelice D, Khiat A, Amara M, Belblidia A, Boulanger Y (2003). MR imaging-guided focused US ablation of breast cancer: histopathologic assessment of effectiveness– initial experience. Radiology.

[18] Zippel DB, Papa MZ (2005). The use of MR imaging guided focused ultrasound in breast cancer patients; a preliminary phase one study and review. Breast Cancer (Tokyo, Japan).

[19] Furusawa H, Namba K, Nakahara H, Tanaka C, Yasuda Y, Hirabara E (2007). The evolving non-surgical ablation of breast cancer: MR guided focused ultrasound (MRgFUS). Breast Cancer (Tokyo, Japan).

[20] Tempany CM, Stewart EA, McDannold N, Quade BJ, Jolesz FA, Hynynen K (2003). MR imaging-guided focused ultrasound surgery of uterine leiomyomas: a feasibility study. Radiology.

[21] Hindley J, Gedroyc WM, Regan L, Stewart E, Tempany C, Hynyen K (2004). MRI guidance of focused ultrasound therapy of uterine fibroids: early results. AJR Am J Roentgenol.

[22] Funaki K, Fukunishi H, Sawada K (2009). Clinical outcomes of magnetic resonance-guided focused ultrasound surgery for uterine myomas: 24-month follow-up. Ultrasound Obstet Gynecol.

[23] Ikink ME, Voogt MJ, Verkooijen HM, Lohle PN, Schweitzer KJ, Franx A (2013). Mid-term clinical efficacy of a volumetric magnetic resonance-guided high-intensity focused ultrasound technique for treatment of symptomatic uterine fibroids. Eur Radiol.

[24] Catane R, Beck A, Inbar Y, Rabin T, Shabshin N, Hengst S (2007). MR-guided focused ultrasound surgery (MRgFUS) for the palliation of pain in patients with bone metastases–preliminary clinical experience. Ann Oncol.

[25] Gianfelice D, Gupta C, Kucharczyk W, Bret P, Havill D, Clemons M (2008). Palliative treatment of painful bone metastases with MR imaging–guided focused ultrasound. Radiology.

[26] Liberman B, Gianfelice D, Inbar Y, Beck A, Rabin T, Shabshin N (2009). Pain palliation in patients with bone metastases using MR-guided focused ultrasound surgery: a multicenter study. Ann Surg Oncol.

[27] Napoli A, Anzidei M, Marincola BC, Brachetti G, Ciolina F, Cartocci G (2013). Primary pain palliation and local tumor control in bone metastases treated with magnetic resonance-guided focused ultrasound. Invest Radiol.

[28] Hurwitz MD, Ghanouni P, Kanaev SV, Iozeffi D, Gianfelice D, Fennessy FM et al. Magnetic resonance-guided focused ultrasound for patients with painful bone metastases: phase III trial results. J Natl Cancer Inst. 2014;106(5). doi:10.1093/jnci/dju082. 10.1093/jnci/dju082 PMC4112926 24760791

[29] Hynynen K, DeYoung D (1988). Temperature elevation at muscle-bone interface during scanned, focused ultrasound hyperthermia. Int J Hyperthermia.

[30] Lutz S, Berk L, Chang E, Chow E, Hahn C, Hoskin P (2011). Palliative radiotherapy for bone metastases: an ASTRO evidence-based guideline. Int J Radiat Oncol Biol Phys.

[31] Huisman M, van den Bosch MA, Wijlemans JW, van Vulpen M, van der Linden YM, Verkooijen HM (2012). Effectiveness of reirradiation for painful bone metastases: a systematic review and meta-analysis. Int J Radiat Oncol Biol Phys.

[32] Mansfield P (1988). Imaging by nuclear magnetic resonance. J Phys E Sci Instrum.

[33] Du J, Hamilton G, Takahashi A, Bydder M, Chung CB (2007). Ultrashort echo time spectroscopic imaging (UTESI) of cortical bone. Soc Magn Reson Med.

[34] De Poorter J, De Wagter C, De Deene Y, Thomsen C, Stahlberg F, Achten E (1995). Noninvasive MRI thermometry with the proton resonance frequency (PRF) method: in vivo results in human muscle. Soc Magn Reson Med.

[35] Ishihara Y, Calderon A, Watanabe H, Okamoto K, Suzuki Y, Kuroda K (1995). A precise and fast temperature mapping using water proton chemical shift. Soc Magn Reson Med.

[36] Huisman M, Lam MK, Bartels LW, Nijenhuis RJ, Moonen CT, Knuttel FM (2014). Feasibility of volumetric MRI-guided high intensity focused ultrasound (MR-HIFU) for painful bone metastases. J Therapeutic Ultrasound.

[37] Kohler MO, Mougenot C, Quesson B, Enholm J, Le Bail B, Laurent C (2009). Volumetric HIFU ablation under 3D guidance of rapid MRI thermometry. Med Phys.

[38] Schenck JF (1996). The role of magnetic susceptibility in magnetic resonance imaging: MRI magnetic compatibility of the first and second kinds. Med Phys.

[39] Perman WH, Moran PR, Moran RA, Bernstein MA (1986). Artifacts from pulsatile flow in MR imaging. J Comput Assist Tomogr.

[40] Peters NH, Bartels LW, Sprinkhuizen SM, Vincken KL, Bakker CJ (2009). Do respiration and cardiac motion induce magnetic field fluctuations in the breast and are there implications for MR thermometry?. J Magn Reson Imaging.

[41] Marieb EN, Koehn K (2007). Human Anatomy and Physiology.

[42] Deckers R, DenisdeSenneville B, Schubert G, Merckel LG, Vaessen HHB, Vanden B (2014). Evaluation of Respiration-Induced Magnetic Field Disturbance Correction of MR Thermometry in Volunteers and in Patients for MR-HIFU Ablation of Breast Cancer: The Effects of Conscious Sedation.

[43] Schmitt A, Mougenot C, Chopra R (2014). Spatiotemporal filtering of MR-temperature artifacts arising from bowel motion during transurethral MR-HIFU. Med Phys.

[44] Staruch R, Chopra R, Hynynen K (2012). Hyperthermia in bone generated with MR imaging-controlled focused ultrasound: control strategies and drug delivery. Radiology.

[45] Vinay R, KusumDevi V. Potential of targeted drug delivery system for the treatment of bone metastasis. Drug Deliv. 2014:1-9. doi:10.3109/10717544.2014.913325. 10.3109/10717544.2014.913325 24839990

[46] Partanen A, Yarmolenko PS, Viitala A, Appanaboyina S, Haemmerich D, Ranjan A (2012). Mild hyperthermia with magnetic resonance-guided high-intensity focused ultrasound for applications in drug delivery. Int J Hyperthermia.

[47] Hey S, Maclair G, de Senneville BD, Lepetit-Coiffe M, Berber Y, Kohler MO (2009). Online correction of respiratory-induced field disturbances for continuous MR-thermometry in the breast. Soc Magn Reson Med.

[48] Wyatt CR, Soher BJ, MacFall JR (2010). Correction of breathing-induced errors in magnetic resonance thermometry of hyperthermia using multiecho field fitting techniques. Med Phys.

[49] Vigen KK, Daniel BL, Pauly JM, Butts K (2003). Triggered, navigated, multi-baseline method for proton resonance frequency temperature mapping with respiratory motion. Soc Magn Reson Med.

[50] Han M, Scott SJ, Ozhinsky E, Salgaonkar V, Larson PEZ, Diederich CJ (2014). Imaging Temperature Changes in Cortical Bone using Ultrashort Echo-time MRI.

[51] Ramsay E, Mougenot C, Kazem M, Laetsch TW, Chopra R. Temperature-dependent MR signals in cortical bone: potential for monitoring temperature changes during high-intensity focused ultrasound treatment in bone. Magnetic resonance in medicine: official journal of the Society of Magnetic Resonance in Medicine / Society of Magnetic Resonance in Medicine. 2014. doi:10.1002/mrm.25492. 10.1002/mrm.25492 PMC4431954 25310966

