# Supplementary figures and images for: Quality of MR thermometry during palliative MR-guided high-intensity focused ultrasound (MR-HIFU) treatment of bone metastases
Source: J Ther Ultrasound. 2015 Mar 24;3:5. doi: 10.1186/s40349-015-0026-7 (PMC4396149; doi:10.1186/s40349-015-0026-7)

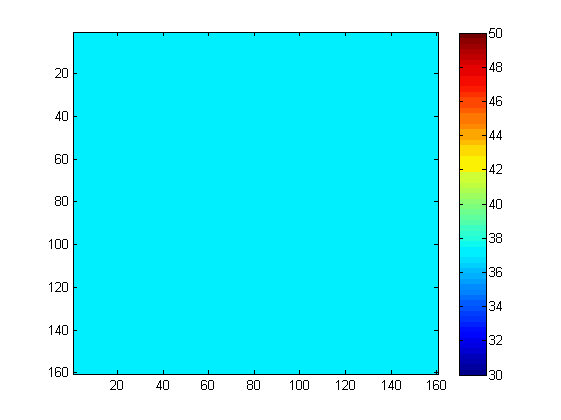

Supplement: Additional file 1: — Movie: typical example of respiratory time-varying field inhomogeneity artifact. To visualize the respiratory time-varying field inhomogeneity artifact more clearly, the dynamic temperature maps of the dataset shown in Figure 4a are shown here as a movie. The typical periodical “blinking” of the temperature map can be observed, most prominently between the 2nd and 3rd frame. Please note that in the top left a non-respiratory time-varying field inhomogeneity artifact is visible, as was pointed out in Figure 4. Also, arterial ghosting artifacts caused by the femoral artery can be seen as a blinking vertical stripe in the middle of the map. [file 40349_2015_26_MOESM1_ESM.gif]
